# Potential benefits of crawl position for prone radiation therapy in breast cancer

**DOI:** 10.1002/acm2.12118

**Published:** 2017-06-26

**Authors:** Bert Boute, Wilfried De Neve, Bruno Speleers, Annick Van Greveling, Christel Monten, Tom Van Hoof, Joris Van de Velde, Leen Paelinck, Werner De Gersem, Tom Vercauteren, Jan Detand, Liv Veldeman

**Affiliations:** ^1^ Faculty of Medicine and Health Sciences Department of Radiotherapy and Experimental Cancer Research Ghent University Ghent Belgium; ^2^ Faculty of Engineering and Architecture Industrial Design Center Ghent University Ghent Belgium; ^3^ Department of Radiation Oncology University Hospital Ghent Ghent Belgium; ^4^ Faculty of Medicine and Health Sciences Department of Anatomy Ghent University Ghent Belgium

**Keywords:** breast cancer, crawl position, prone radiotherapy

## Abstract

**Purpose:**

To investigate crawl position with the arm at the treated side alongside the body and at the opposite side above the head for prone treatment in patients requiring breast and regional lymph node irradiation.

**Methods:**

Patient support devices for crawl position were built for CT simulation and treatment. An asymmetric fork design resulted from an iterative process of prototype construction and testing. The fork's large horn supports the hemi‐thorax, shoulder, and elevated arm at the nontreated side and the head. The short, narrow horn supports the arm at the treated side. Between both horns, the treated breast and its regional lymph nodes are exposed. Endpoints were pain, comfort, set‐up precision, beam access to the breast and lymph nodes, and plan dose metrics. Pain and comfort were tested by volunteers (*n *= 9); set‐up precision, beam access, and plan dose metrics were tested by means of a patient study (*n *= 10). The AIO™ (Orfit, Wijnegem, Belgium) prone breastboard (AIO™) was used as a reference regarding comfort and set‐up precision.

**Results:**

Pain at the sternum, the ipsilateral shoulder, upper arm, and neck was lower in crawl position than with bilateral arm elevation on AIO™. Comfort and set‐up precision were better on the crawl prototype than on AIO™. In crawl position, beam directions in the coronal and near‐sagittal planes have access to the breast or regional lymph nodes without traversing device components. Plan comparison between supine and crawl positions showed better dose homogeneity for the breast and lymph node targets and dose reductions to all organs at risk for crawl position.

**Conclusions:**

Radiation therapy for breast and regional lymph nodes in crawl position is feasible. Good comfort and set‐up precision were demonstrated. Planning results support the hypothesis that breast and regional lymph nodes can be treated in crawl position with less dose to organs at risk and equal or better dose distribution in the target volumes than in supine position. The crawl technique is a candidate methodology for further investigation for patients requiring breast and regional lymph node irradiation.

## 1 INTRODUCTION

Radiotherapy after breast‐conserving surgery improves loco‐regional control and survival at the expense of acute and late toxicity to the treated region, radiation‐induced cardiac events, lung cancer, and cancer in the nontreated breast.^1^, ^2^, ^3^, ^4^, ^5^, ^6^ Prone radiotherapy allows decreasing acute toxicity, cosmetic changes, risks of radiation‐induced lung cancer, and cardiac toxicity.^7^, ^8^, ^9^ However, several drawbacks of prone position are reported, including reduced set‐up precision and discomfort.^10^

Two classes of patient support devices for prone radiotherapy can be distinguished. Prone breastboards rest entirely on the treatment couch surface while prone breastcouches replace the couch blade (or its cranial part) so that no couch parts extend below the treated breast. Commercial devices of both classes are designed to support the patient with both arms elevated. The arm position, the treatment couch when using breastboards, as well as device components that support the elevated arm at the treated side are in the way of anterior beam directions for breast and lymph node irradiation (B+LNI).

Although clinical experience using posterior beams to treat axillary and periclavicular lymph node chains has been described,^11^ prone radiotherapy is rarely used in patients requiring B+LNI. This is unfortunate because patients requiring B+LNI receive substantially more lung dose than patients treated with breast irradiation only, due to irradiation of the lung top nearby the axillary and periclavicular lymph node regions. The correlation between lung dose and death due to second primary lung cancer is well documented.^3^, ^4^, ^6^ Irradiation of the internal mammary chain increases heart dose and was shown to increase the rate of major cardiac events.^5^, ^6^, ^12^ Hence, risk for radiation‐induced heart disease or lung cancer induction might be reduced if heart and lung dose could be decreased by using prone B+LNI.

With reduction in lung and heart dose in the setting of B+LNI as main objective, we investigated a new prone position with the arm at the treated side alongside the body and the arm at the contralateral side above the head, further called crawl position because it resembles a phase of crawl swimming. Support devices for crawl position were built as breastboards (for CT simulation) and breastcouches (for treatment). Comparative assessment was performed with prone bilateral arm elevation regarding feasibility, comfort, and set‐up precision and with supine position regarding dose to targets and organs at risk in B+LNI.

## 2 MATERIALS AND METHODS

**Figure 1:**
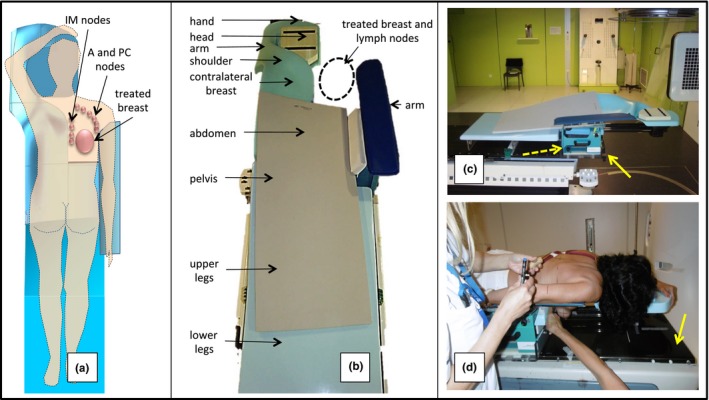
(a) Asymmetric fork shape of the crawl positioning device visible from above through the semitransparent drawing of a patient. Projection of the right breast and its regional node chains in relation to the aperture. (b) Photograph from above the caudal end of the crawl breastcouch for right‐side irradiation. Arrows indicate roughly the position of different body regions when an average‐size patient is positioned on the device. The grey material is a foam mat used for patient comfort. (c) Photograph from the right side of the device mounted on the I‐beam EVO of the linear accelerator as a breastcouch. The solid yellow arrow indicates the cranial end of the linear accelerator couch. The dotted yellow arrow indicated the pedestal that allows adjusting the arm support (5 degrees of freedom: 3 translations + pitch and yaw) to the patient's anatomy. (d) Photograph of a patient laying on the device mounted as a breastboard on the couch blade (indicated by yellow arrow) of the CT simulator.

To test crawl position, we constructed prone breastboards and ‐couches with an upper surface that supported the entire body except the treated breast, and the ventral body regions overlaying the axillary, periclavicular and internal mammary lymph node regions. Above the waist, the resulting support surface is shaped as an asymmetric fork [Fig. 1(a)] with a short, narrow horn supporting the arm at the treated side and a large horn supporting the hemi‐thorax, breast, shoulder, and elevated arm at the nontreated side as well as the head [Fig. 1(b)]. The treated breast and its regional lymph nodes is positioned between both horns. The device is mounted on the caudal part of an I‐Beam EVO couch blade of an Elekta Synergy linear accelerator [Fig. 1(c)]; the cranial part being removed. Hence, the device is mounted as a crawl breastcouch with no parts of the I‐beam EVO couch blade below the fork horns. The crawl breastcouch is used with a floor laser which projects a longitudinal laser line directly on the breast and shoulder of patients laying on the crawl device. The floor laser is used for left‐right positioning. The standard lateral lasers are used for longitudinal and height positioning of patients. On the CT simulator, the device is placed as a crawl breastboard on the CT‐couch blade [Fig. 1(d)]. Hence, a floor laser cannot be used. Lateral laser set‐up marks are drawn during CT simulation as well as a longitudinal laser line mark on the back of the patient. Before the first treatment, these longitudinal laser marks are used for set‐up and cone‐beam CT‐based adjustment of the set‐up is performed using the simulator CT as reference. The floor laser line is delineated on the patient's breast and periclavicular skin. Floor and lateral lasers are used for set‐up during subsequent session.

Nine volunteers (female personnel and ex‐patients) with various anatomy were selected for comfort and pain assessment using questionnaires. All volunteers were familiar with the AIO™ breastboard. Volunteers were positioned for both left breast irradiation with bilateral arm elevation using the modified AIO™ (Orfit, Wijnegem, Belgium) prone breastboard (AIO™)^7^ and in crawl position using the new device. They were asked to lie immobile for 10 min. Subsequently the questionnaire was given. Six body regions (neck, left‐/right shoulder, sternum, and left‐/right arm) could be rated on a visual analogue scale from 0 to 10 going from no pain reported to an unbearable pain experienced, respectively. Comfort and pain scores of both devices were compared.

One patient who was eligible for B+LNI was CT scanned in crawl and in standard supine position. The clinical target volume (CTV) consisted of the whole left breast, the left axillary levels I‐III ,and the left supra‐ and infra‐clavicular lymph node regions. CTV of the whole breast was delineated in prone and supine positions as described previously.^7^ CTV of axillary and periclavicular lymph node regions was delineated according to the PROCAB guidelines (http://www.abro-bvro.be/index.php?option=com_content&view=category&id=94&Itemid=940).^13^

Ten patients received half of their WBI treatment sessions on the crawl breastcouch and the other half on AIO™. The patients were selected as follows: female, 45 years or older, right‐sided breast carcinoma, suitable for adjuvant radiotherapy after lumpectomy for breast cancer, and prone WB irradiation without LNI.

Laser‐based set‐up was performed in both positions and set‐up errors were measured using daily cone‐beam CT scanning as reference.^10^, ^14^ The magnitude of set‐up errors was calculated as described before.^10^

All studies were approved by the ethics committee of Ghent University Hospital and informed consent was obtained from the study participants.

## 3 RESULTS

Comfort was optimized by an iterative process of prototype construction, testing, and redesign. AIO™ served as a reference. We report results obtained on AIO™ and on the crawl breastboard (prototype version shown in Fig. 1(d) which is presently used in clinical trials).

Ex‐patients positioned on AIO™ reported discomfort caused by bilateral arm elevation and had to exert force by the arm at the operated side to maintain a stable position. The ipsilateral arm support of the crawl prototypes provides stability by preventing lateral and downward movement. The arm alongside the body was reported to be more comfortable than the elevated arm position, especially after axillary node dissection.

**Figure 2:**
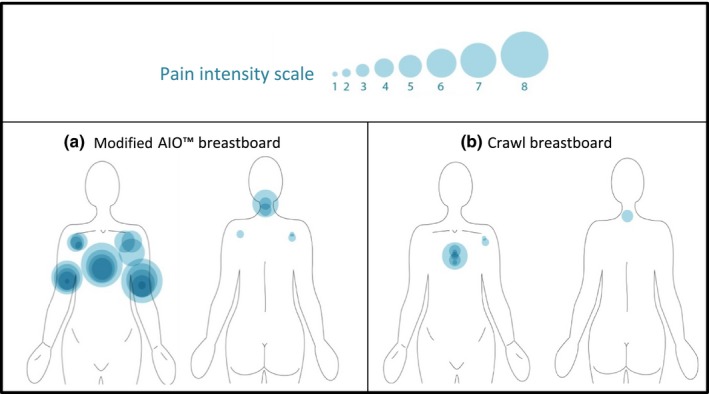
Pain intensity scale: each circle represents a painful region reported by one volunteer. The size of the circle increases with severity of the pain. Pain scored for left‐side irradiation set‐up on AIO™ (a) or on the crawl breastboard (b).

Pain was scored by nine volunteers (Fig. 2). On the AIO™, pain was frequently reported at the sternum near the edge of the surface supporting the nontreated breast; at the ipsilateral shoulder, at both upper arms, and at the neck [Fig. 2(a)]. On the crawl breastboard, sternal pain was reported less frequently and was less severe [Fig. 2(b)]. Pressure on the sternum can be lowered by raising the ipsilateral arm and shoulder support. A minor pain point was reported at the edge of the arm support at the ipsilateral side. On AIO™, pain at the anterior and medial side of the ipsilateral upper arm seems caused by arm elevation and muscle contraction to maintain stability. Similar pain was not reported using crawl prototypes. Neck pain was mild or absent on crawl prototypes.

**Figure 3:**
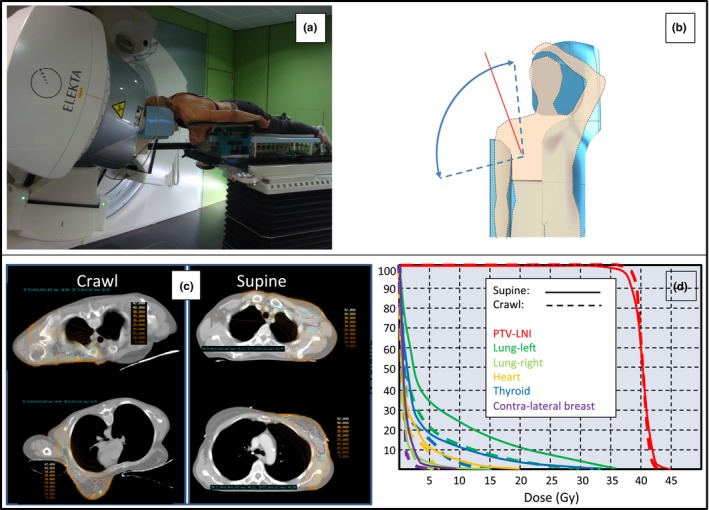
(a) Illustration of a gantry −80°, couch isocenter −70° beam direction (Elekta coordinate system). (b) Unobstructed beam access range to the breast or lymph node targets exceeds 90° in the coronal plane (sector between dotted blue lines) and 180° in a near‐sagittal plane (indicated by a red line) at a couch isocenter rotation of ~|70°|. (c) and (d) Dose distributions and dose‐volume histograms (DVH) of a patient eligible for B+LNI using prone crawl radiotherapy (left images in [c]; dotted lines in [d]) or standard supine radiotherapy (right images in [c]; solid lines in [d]). PTV‐LNI = planning target volume for lymph node irradiation.

Figure 3(a) gives an impression of the exposure of the ipsilateral breast and shoulder. It illustrates a left posterior‐superior oblique beam direction with couch isocenter −70° (near‐sagittal) and gantry −80° in the Elekta coordinate system. Beam directions in the near‐sagittal plane (Fig. 3(b): plane illustrated by the red line and obtained by a |70°| couch isocenter rotation) were used to obtain the plan in crawl position for B+LNI. Figure 3(c) shows the planning comparison between standard supine and crawl positions using multibeam IMRT in the setting of a B+LNI at the left side. The plan in crawl position yielded better dose homogeneity for the breast (not shown) and lymph node targets as well as dose reductions to all organs at risk [Fig. 3(d)]. Using the crawl breastcouch and the floor laser, the random set‐up error in the left‐right direction was less than 3 mm in nine of ten patients and was 4 mm in the 10^th^ patient. On AIO™, nine of ten patients had a random set‐up error of more than 3 mm (>5 mm in 5 patients; >8 mm in 3 patients). The difference is significant (*P* = 0.013, paired student T test). Random set‐up errors were equal for the crawl breastcouch and AIO™ in the antero‐posterior or cranio‐caudal directions.

## 4 DISCUSSION

A review of the advantages, disadvantages, challenges, limitations of prone position for breast irradiation has been published by the investigators at New York University.^15^ Prone radiotherapy is advantageous for the vast majority of patients requiring breast irradiation irrespective of breast size: lower dose to lung and heart, less acute toxicity, and better cosmesis.^7^, ^8^, ^9^, ^15^ Challenges of prone radiotherapy are numerous^15^ and centers that wish acquiring prone breast radiotherapy face a substantial learning effort. The drawbacks and the learning effort necessary to acquire prone breast radiotherapy may explain the adherence to supine breast cancer radiotherapy in the vast majority of centers worldwide. We investigated prone radiotherapy for breast cancer since 2008, first using the Horizon prone breastboard (Civco Medical Solutions, Orange City, Iowa, USA)^13^ and later the AIO™ breastboard which we modified^7^ to become the device that we use in clinical practice. With AIO™, no patients requiring B+LNI (about three of ten patients referred to our centre require B+LNI) were treated in prone position because of the restrictions regarding good beam directions. The aim of crawl positioning research was to offer a prone solution for these patient groups.

We generated proof that crawl position is a feasible alternative for prone position with bilateral arm elevation. We were able to construct prototypes that showed better patient comfort than the two commercial prone breastboards (Horizon and AIO™) that we used until now. We demonstrated that crawl position on properly designed devices allows a large, unobstructed beam access range to the breast and its regional lymph node regions (Fig. 3, panel B). Planning results confirm the hypothesis that crawl position may offer a solution for B+LNI with considerable reduction in lung and heart dose as compared to supine B+LNI. When using beam directions in the near‐sagittal plane, heart and lung dose reduction can be achieved without increased doses to the contralateral breast or thyroid. Crawl breastcouches allow the use of a floor laser to enhance set‐up precision in the left‐right direction.

Most commercial prone breastboards or ‐couches have a basic left‐right symmetric design. Relatively inexpensive add‐on components are used to obtain a configuration for left or right breast treatment. We abandoned this concept early on the drawing table because of the large left‐right asymmetry of the crawl position. Left‐ and right‐side‐specific devices were built. Efficient clinical practice requires a set of four crawl devices: left and right devices that are used as boards on the CT simulator and as couches on the treatment machine. Hence, investment cost may be a concern.

The prone crawl position seems promising, but several challenges and limitations of the crawl breastcouch prototype hamper its widespread use. First, the prototype is the result of an iterative process of tests and improvements which resulted in using many components and materials which makes it unsuitable for modern industrial production. Furthermore, many components were overdimensioned to avoid the need for stress testing with the drawback of adding weight (~17 kg for the complete device). An in silico study to reduce the number of components and using lightweight materials is work in progress. Carbon fiber will replace fiberglass, polycarbonate, or polymethylmethacrylate. A substantial weight loss should be possible by replacing the folded steel plate arm support base by lightweight material. The second objective of the in silico study is to make a crawl breastcouch which is MRI compatible. Clinical challenges were encountered in obese patients. Abdominal fat was pushed cranially over the edge of the abdominal support surface into the aperture for the treated breast and arm fat bulged between the medial edge of the arm support blade and the lateral thoracic wall near the treated breast. We address such problems by fabricating patient‐specific garments. One piece consists of the unilateral breast holder^7^ to which a corset is knitted using a computer‐controlled knitting machine [W. De Neve, unpublished]. The other piece consists of a computer‐knitted sleeve in resilient material [W. De Neve, unpublished]. Fabrication is performed and financed by a company specialized in medical garments for patients with severe burns (Tricolast, Deinze, Belgium).

To date, we have treated 50 patients in crawl position. The prone deep inspiration breath hold technique^9^ was easily adapted to the crawl breastboards and ‐couches (L. Veldeman, unpublished). All results to date support the hypothesis that crawl breast cancer radiotherapy is a candidate technique to reduce the long‐term risk of radiation‐induced lung cancer induction and cardiac injury. Randomized trials comparing crawl with supine position in the B+LNI setting are in preparation.

## 5 SUMMARY

Crawl position, with the arm at the treated side alongside the body and at the opposite side above the head, was investigated for prone breast cancer radiation therapy. As compared to the commonly used prone position with bilateral arm elevation, crawl position shows better comfort, stability, and set‐up precision and permits a vast range of beam directions in the coronal and near‐sagittal planes that reach the breast and regional lymph nodes without passage through components of the crawl positioning device. Near‐sagittal beam directions seem valuable to reduce dose to heart, lung, and contralateral breast in patients who require irradiation of regional lymph nodes.

## CONFLICT OF INTEREST

Ghent University owns the patent application entitled Radiotherapy Board and Couch [WO2015144654A1] filed on 25.03.2014 for which Wilfried De Neve, Bruno Speleers, Bert Boute, and Liv Veldeman own the intellectual rights.
